# *Sinosasa subglabra* (Poaceae, Bambusoideae), a new bamboo species from Guangxi, China

**DOI:** 10.3897/phytokeys.281.213873

**Published:** 2026-11-06

**Authors:** Zhuo-Yu Cai, Jin-Wei Fu, Hai-Xiang Yuan

**Affiliations:** 1 State Key Laboratory for Development and Utilization of Forest Food Resources, Nanjing Forestry University, Nanjing 210037, China State Key Laboratory for Development and Utilization of Forest Food Resources, Nanjing Forestry University Nanjing China; 2 Co-Innovation Center for Sustainable Forestry in Southern China, Nanjing Forestry University, Nanjing 210037, China Co-Innovation Center for Sustainable Forestry in Southern China, Nanjing Forestry University Nanjing China; 3 Bamboo Research Institute, Nanjing Forestry University, Nanjing 210037, China Bamboo Research Institute, Nanjing Forestry University Nanjing China

**Keywords:** Arundinarieae, morphology, taxonomy, woody bamboo

## Abstract

A new bamboo species, *Sinosasa subglabra*, from Guilin City, Guangxi Province, China, is described and illustrated here. The new species resembles *Sinosasa huapingensis* but can be easily distinguished by its glabrous or pubescent internode surfaces, glabrous culm leaf abaxial surfaces except for the white pubescence at the base, 3–6 foliage leaves on ultimate branches, many 5–10(–13) mm long fimbriae on foliage leaf auricles, and glabrous foliage leaf abaxial surfaces.

## Introduction

*Sinosasa* L.C. Chia ex N.H. Xia, Q.M. Qin & Y.H. Tong is a shrubby bamboo genus endemic to subtropical areas of China (Qin et al. 2021). It belongs to the tribe Arundinarieae (Poaceae, Bambusoideae) and currently includes nine species (Zhou et al. 2026). Phylogenetic studies have strongly supported the monophyly of *Sinosasa* based on both plastid and nuclear data (Zeng et al. 2010; Zhang et al. 2012; Qin et al. 2021; Li et al. 2023; Zhang et al. 2025). Since its formal establishment with seven species, *Sinosasa* has expanded through continued field investigations and taxonomic studies. Subsequent studies transferred previously misplaced taxa into the genus, including *Sinosasa gracilis* (B.M. Yang) N.H. Xia, Y.H. Tong, J.B. Ni & X. Li and *Sinosasa damingshanensis* (Hsueh & W.P. Zhang) N.H. Xia & Y.L. Ding (Li et al. 2023; Zhang et al. 2025). *Sinosasa* features a suite of distinctive vegetative characters: leptomorph rhizomes, pluricaespitose culms, strongly raised supranodal ridges, a solitary branch at each node, and especially very long foliage leaf ligules (except for *S. gracilis*), which make it easy to identify in the field (Qin et al. 2021; Li et al. 2023; Zhang et al. 2025). On the other hand, the genus is reproductively characterized by true spikelets, each spikelet containing 2–3 perfect florets, with each floret possessing three stamens and two stigmas (Qin et al. 2021).

During field surveys in Lingchuan County, Guilin City, Guangxi Province, we encountered an unknown running bamboo. Although it was not flowering, its vegetative characters were sufficient to determine its generic placement. This bamboo possesses the characters: pluricaespitose culms, strongly raised culm supranodal ridges, and a solitary branch at each node, which conform to the diagnostic characters of *Sinosasa* (Qin et al. 2021). After morphological comparison with all previously recorded species of *Sinosasa* (Qin et al. 2021; Li et al. 2023; Zhang et al. 2025), we concluded that this species is new to science. Therefore, a formal description along with an illustration is provided below.

## Materials and methods

Specimens of the new species were collected from the type locality in 2025 and 2026. Voucher specimens were deposited in the Herbarium of Nanjing Forestry University (NF). Herbarium acronyms follow Index Herbariorum (2026). Morphological comparison was based on relevant literature (Qin et al. 2021; Li et al. 2023; Zhang et al. 2025), specimens, and living plants. Both the type specimen and photographs were used to prepare the description. Terminology follows Li et al. (2006) and Beentje (2016).

## Taxonomic treatment

### Sinosasa subglabra

Taxon classificationPlantaePoalesPoaceae

Z.Y.Cai sp. nov.

CC269953-8046-5120-8B16-9FE9D9135A18

urn:lsid:ipni.org:names:77495563-1

Fig. 1

#### Type.

China • Guangxi Province: Guilin City, Lingchuan County, Dajing Town, Xinzhai Village, Qifen Mountain, 25°04'40.75"N, 110°36'35.77"E, elev. 1,057 m, 12 June 2025, *Z.Y. Cai & X. Zhang CZYNB-18* (Holotype: NF!).

**Figure 1.**
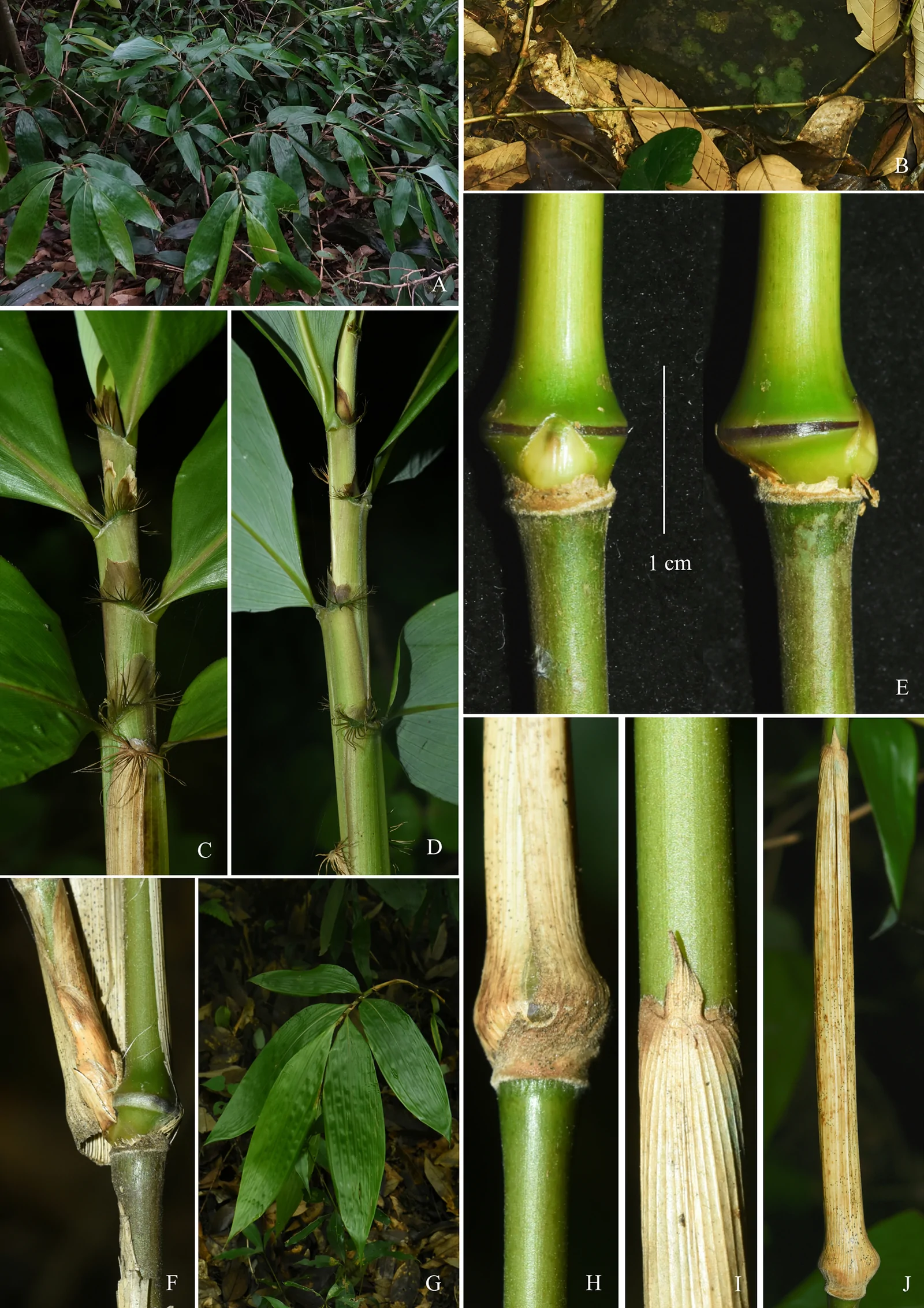
**A**. Habit; **B**. Rhizomes; **C**. Foliage leaf auricles on the adaxial side; **D**. Foliage leaf auricles on the abaxial side; **E**. Primary branch buds and dark green supranodal ridges; **F**. Branch complement; **G**. Leafy branch; **H**. Lower part of culm leaf; **I**. Upper part of culm leaf; **J**. Culm leaf. Photos by Zhuo-Yu Cai.

#### Diagnosis.

*Sinosasa subglabra* is morphologically similar to *Sinosasa huapingensis* but differs by its glabrous or pubescent internode surfaces, glabrous except for base white pubescent culm leaf abaxial surfaces, 3–6 foliage leaves on ultimate branches, many 5–10(–13) mm long fimbriae on foliage leaf auricles, glabrous foliage leaf abaxial surfaces (Table 1).

**Table 1.** Morphological comparison of *Sinosasa subglabra* and *Sinosasa huapingensis*.

**Characters**	***S. subglabra***	***S. huapingensis***
Internode indumentum	Glabrous or pubescent	Upper part deciduously sparsely brown hirsute
Culm leaf		
upper part indumentum of sheath	Glabrous	Sparsely brown strigose
base indumentum of sheath	White pubescent	Densely brown hirsute
Foliage leaf		
Leaf number on each ultimate branch	3–6	8–17
Fimbriae number on auricles	Many	2–4
Fimbriae length	5–10(–13) mm	2–6 mm
Blade size	(10–)20–30 cm long (3–)6–7.5 cm wide	20–25 cm long 3.5–5.5 cm wide
Abaxial surface indumentum of blades	Glabrous	Puberulent

#### Description.

Shrubby bamboo; rhizomes leptomorph, culms pluricaespitose. Culms erect, 1–1.5 m tall, ca. 0.5 cm in diameter; internodes green, terete, glabrous or pubescent; supranodal ridges dark green, strongly raised, sheath scars white pubescent; branch complements with solitary branch; primary culm buds triangular to lanceolate, glabrous, margins pubescent. Culm leaves persistent, papery, ca. 1/2 as long as internodes, abaxial surfaces glabrous except for base white pubescent, margins glabrous; culm leaf auricles and fimbriae absent; culm leaf ligules truncate or slightly retuse, ca. 1 mm high, margins puberulent; culm leaf blades small, lanceolate. Ultimate branches with 3–6 foliage leaves; foliage leaf sheaths pubescent, margins glabrous; foliage leaf auricles small, 0.6–0.8 mm long, margins with many spreading fimbriae, fimbriae 5–10(–13) mm long; foliage leaf ligules conspicuous, 1–1.5 cm high, apex arched, glabrous; leaf blades large, lanceolate to oblong-elliptic, thick papery, wavy when dry, (10–)20–30 cm long, (3–)6–7.5 cm wide, apex long acuminate, base attenuate; both surfaces glabrous; longitudinal veins 5–9 pairs, margins serrulate on one side, entire on the other. Flowers and fruits unknown.

#### Phenology.

Shooting in May.

#### Distribution and habitat.

This new species has only been found at its type locality so far. It occurs on mountain slopes at an elevation of approximately 1,000 m.

#### Etymology.

The specific epithet refers to the culm leaf abaxial surfaces, which are glabrous except for white pubescence at the base, while the abaxial surfaces of most other congeneric species are strigose.

#### Chinese name.

少毛华赤竹 (Chinese Pinyin: shăo máo huá chì zhú).

#### Additional specimens examined.

China • Guangxi Province: Guilin City, Lingchuan County, Dajing Town, Xinzhai Village, Qifen Mountain, elev. 1,057 m, 15 June 2026, *Z.Y. Cai, J.W. Fu & H.X. Yuan CZYNB-43* (Paratype: NF!).

## Supplementary Material

XML Treatment for Sinosasa subglabra

## Additional information

### Artificial Intelligence (AI) use

The authors accept full responsibility for the content of the manuscript, including the disclosure of any use of AI.

No AI tools were used in the preparation of this manuscript.

### Author ORCIDs

Z.-Y. Cai https://orcid.org/0000-0001-9288-0882

J.-W. Fu https://orcid.org/0009-0000-5738-0276

H.-X. Yuan https://orcid.org/0009-0005-0385-5527

### Data availability

All of the data that support the findings of this study are available in the main text.
